# Anaesthetic Effects of Eugenol on Grass Shrimp (*Palaemonetes sinensis*) of Different Sizes at Different Concentrations and Temperatures

**DOI:** 10.1038/s41598-018-28975-w

**Published:** 2018-07-20

**Authors:** Yingdong Li, Qiuxin She, Zhibin Han, Na Sun, Xu Liu, Xiaodong Li

**Affiliations:** 1 College of Animal Science and Veterinary Medicine, Shenyang Agricultural University, 110866 Shenyang, China; 2 Panjin Guanghe Fisheries Co. Ltd, 124200 Panjin, China

## Abstract

Essential oil derivatives are widely used for anaesthetising aquatic animals. However, the effectiveness of anaesthesia often varies according to the anaesthetic agent, species, temperature, dosage, and interactions among these factors. This study evaluated the effects of eugenol on three sizes of the shrimp *Palaemonetes sinensis* at different concentrations and temperatures. Eugenol dose, water temperature, and shrimp size were found to significantly influence anaesthesia in *P. sinensis*. Induction time decreased linearly with increasing water temperature and eugenol concentration, while it increased with body weight. However, recovery times lengthened with increasing concentration and temperature, and shortened with lower body size. At 100 and 200 μL/L eugenol concentrations, the survival rates of medium and large shrimps were maintained at over 80% at all temperatures studied over 72 h recovery. However, the survival rates of small shrimps were below 60% at 24 °C and 28 °C over 5 days of recovery. These results suggest that eugenol is an effective and rapid anaesthetic for *P. sinensis*, but it might have disadvantages such as slow recovery and possible mortality in small shrimps and at higher temperatures and dosages.

## Introduction

Anaesthetics are widely used to reduce physiological stress and mechanical damage in aquaculture and fisheries during netting, handling, sampling, and transportation^1–3^. Anaesthetics increase the survival rate and are an important welfare tool during such activities^4^. A variety of anaesthetic agents are used in aquaculture, including tricaine methanesulphonate (MS-222), 2-phenoxyethanol, quinaldine, benzocaine, Aqui-S^TM^, and metomidate^5–9^, but most of these pose some degree of toxicity to humans and the environment. The only anaesthetic approved by the US Food and Drug Administration for fish is MS-222, which is expensive and requires at least a 21-day recovery period before treated fish can be used for human consumption^10^. In addition, MS-222 is not effective for most cultured crustacean species^11^.

Eugenol, the essential oil from cloves, has been found to be relatively safe, efficacious, and inexpensive for anaesthetising the crustacean species *Homarus americanus*^12^, *Macrobrachium rosenbergii*^1^, *Fenneropenaeus indicus*^13^, and *Macrobrachium tenellum*^14^. However, the effectiveness of anaesthetics often varies according to species, age, weight, temperature, dose, and interactions among these factors. Although some research has shown that these environmental factors mght affect eugenol anaesthesia efficiency in fish, these considerations have not yet been explored in crustaceans. Moreover, our previous study demonstrated that menthol dose, water temperature and shrimp size significantly influence anaesthesia and survival rate in *P. sinensis*^15^.

The Chinese grass shrimp (*Palaemonetes sinensis*; Sollaud, 1911) is a 2–5 cm species belonging to the Palaemonidae family of decapod crustaceans, and it is widely distributed in China and adjacent areas^16^. *P. sinensis* has a long history of human consumption in China and often draws higher market prices than those of penaeid species. Furthermore, because of its striking appearance, live *P. sinensis* is often traded for aquarium purposes or as bait for sport fishing, especially in Japan^17^. Although *P. sinensis* is a slow-swimming shrimp and is easy to handle, it can suffer pain and stress during capture, crowding, handling, and transport, which can lead to high morbidity and mortality. The aim of the present study was to analyse the anaesthetic effectiveness of eugenol on *P. sinensis*. In addition, the effects of water temperature and body weight on the efficacy of anaesthetics were also tested.

## Results

### Induction time

**Figure 1:**
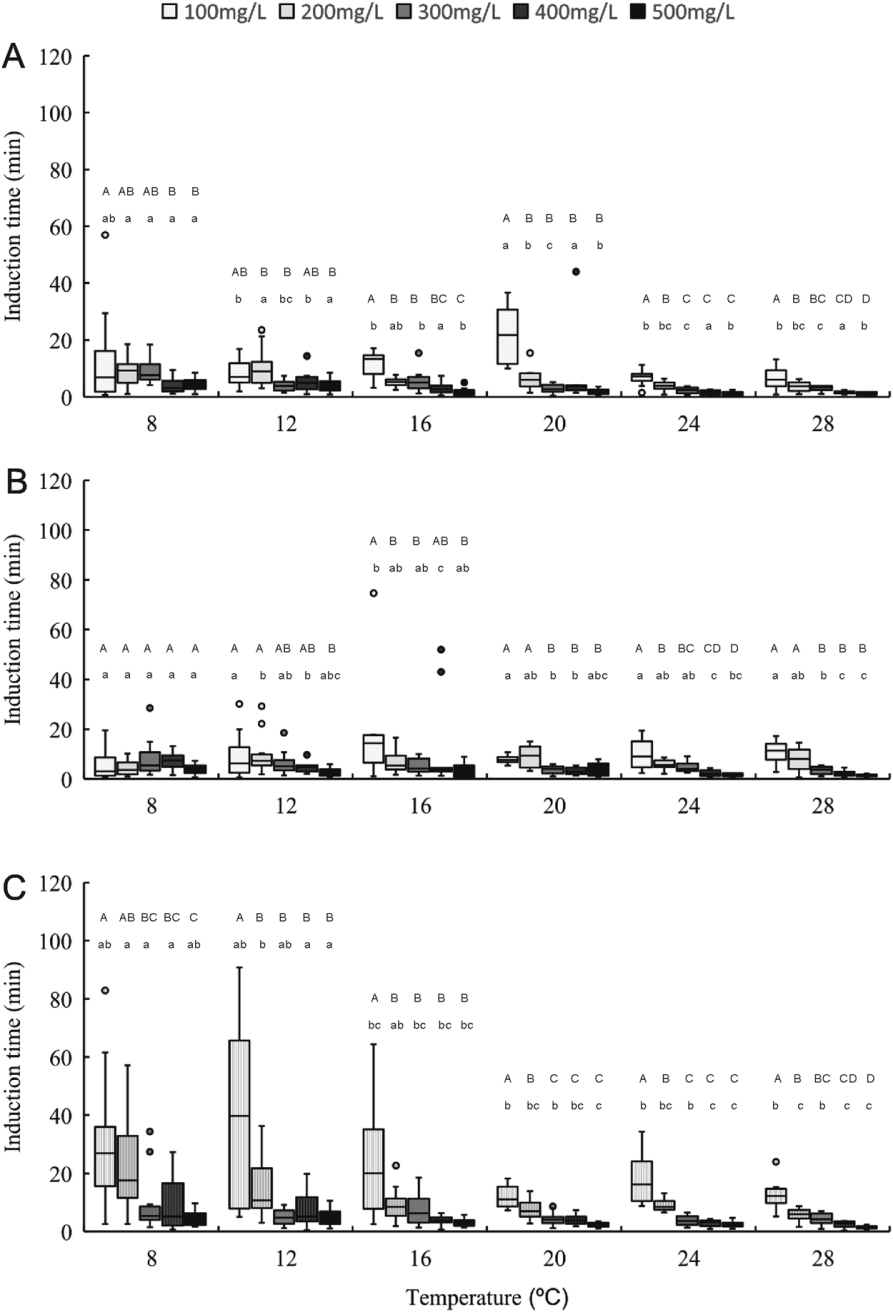
The time required for anaesthetic induciton stage 1 in different eugenol concentritiaons and tempertures through small (**A**), normal (**B**) and large (**C**) shrimps. One-way ANOVA showed significant (*P* < 0.05) temperature and dose effects for each size of *Palaemonetes sinensis*. Lowercase letters indicate significant differences within a temperature. Uppercase letters indicate significant differences within a dose.

**Figure 2:**
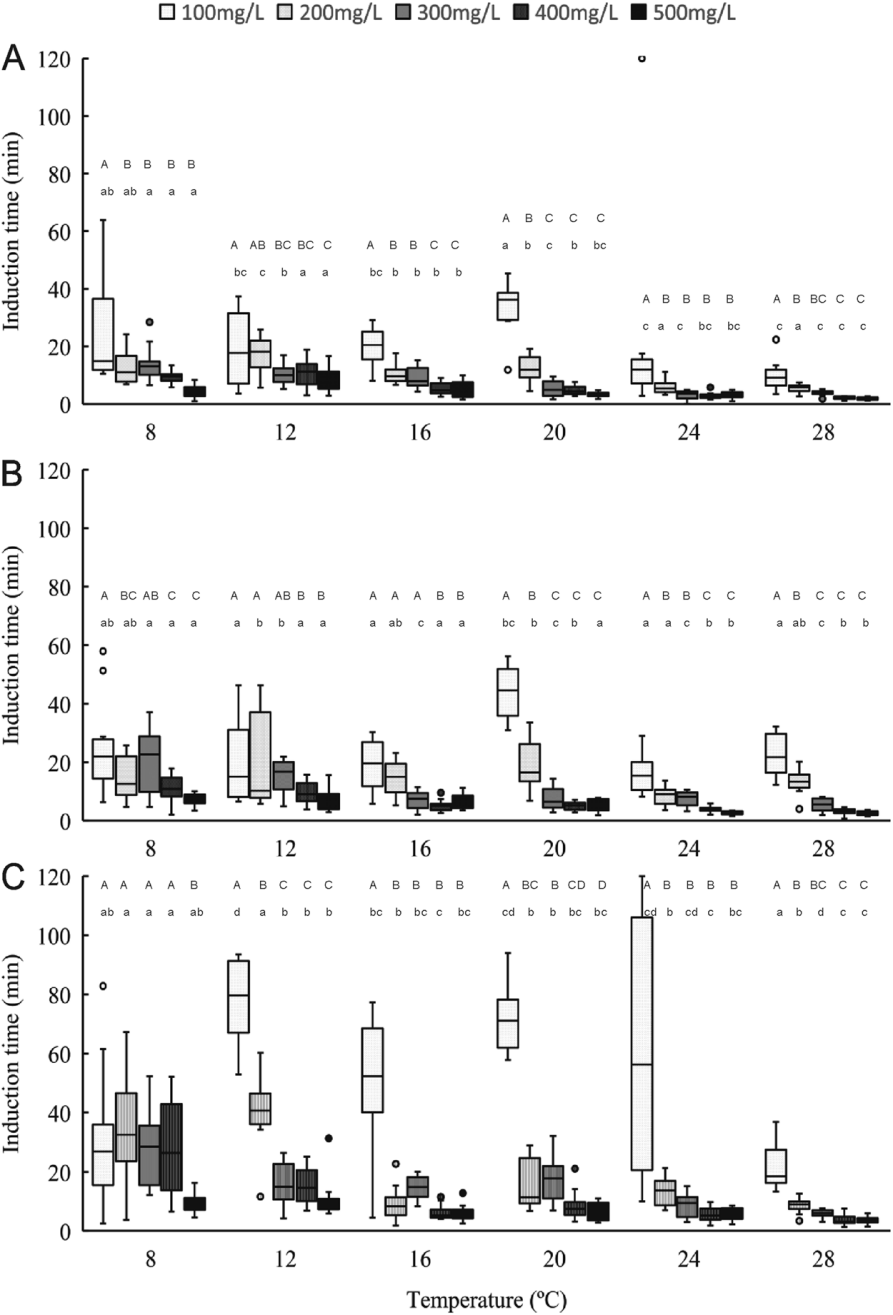
The time required for anaesthetic induciton stage 2 in different eugenol concentritiaons and tempertures through small (**A**), normal (**B**) and large (**C**) shrimps. One-way ANOVA showed significant (*P* < 0.05) temperature and dose effects for each size of *Palaemonetes sinensis*. Lowercase letters indicate significant differences within a temperature. Uppercase letters indicate significant differences within a dose.

The induction time data for anaesthesia stage 1 at all sizes, temperatures, and eugenol concentrations are shown in Fig. 1. Water temperature and eugenol concentration significantly affected induction time (*P* < 0.05), with induction time decreasing linearly as the concentration and temperature increased. Eugenol at 100 μL/L concentration yielded slower induction than did the 400 μL/L and 500 μL/L concentrations at all temperatures and across all size classes. The 8 °C and 12 °C temperature groups showed slower induction times than did the groups at 24 °C and 28 °C, at all concentrations and sizes. Shrimps of all sizes exposed to all concentrations of eugenol and temperatures reached induction stage 1 in under 60 min. At temperatures between 12 °C and 24 °C, the induction time to stage 2 anaesthesia for large shrimps was more variable at 100 μL/L than at other concentrations(Fig. 2). Induction times for stages 1 and 2 were rapid and similar across all groups at 300–500 μL/L eugenol at temperatures above 16 °C. These results indicate that temperature and dose had a statistically significant interaction in affecting induction time (Table S1).

### Time to recovery

**Figure 3:**
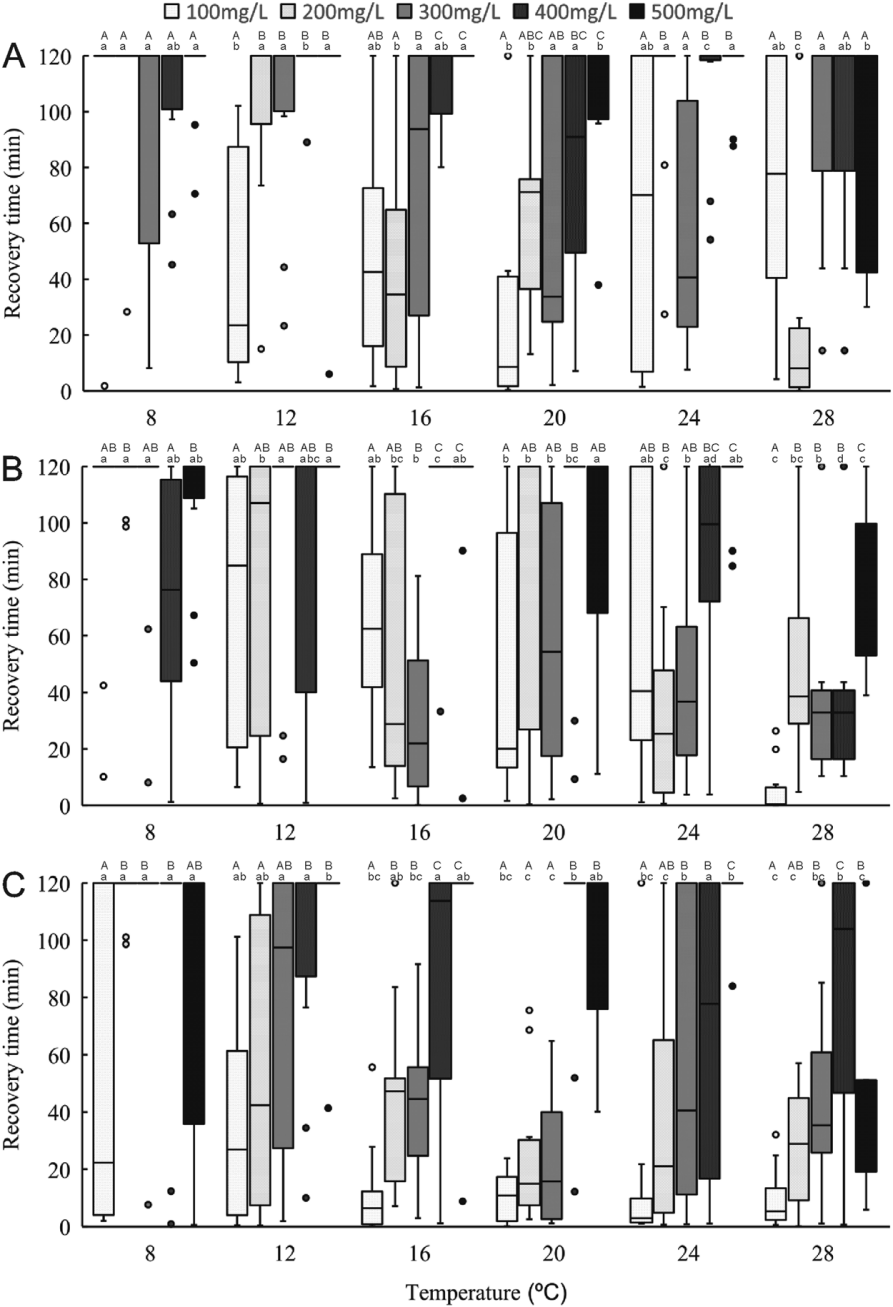
The time required for anaesthetic recovery stage 1 in different eugenol concentritiaons and tempertures through small (**A**), normal (**B**) and large (**C**) shrimps. One-way ANOVA showed significant (*P* < 0.05) temperature and dose effects for each size of *Palaemonetes sinensis*. Lowercase letters indicate significant differences within a temperature. Uppercase letters indicate significant differences within a dose.

**Figure 4:**
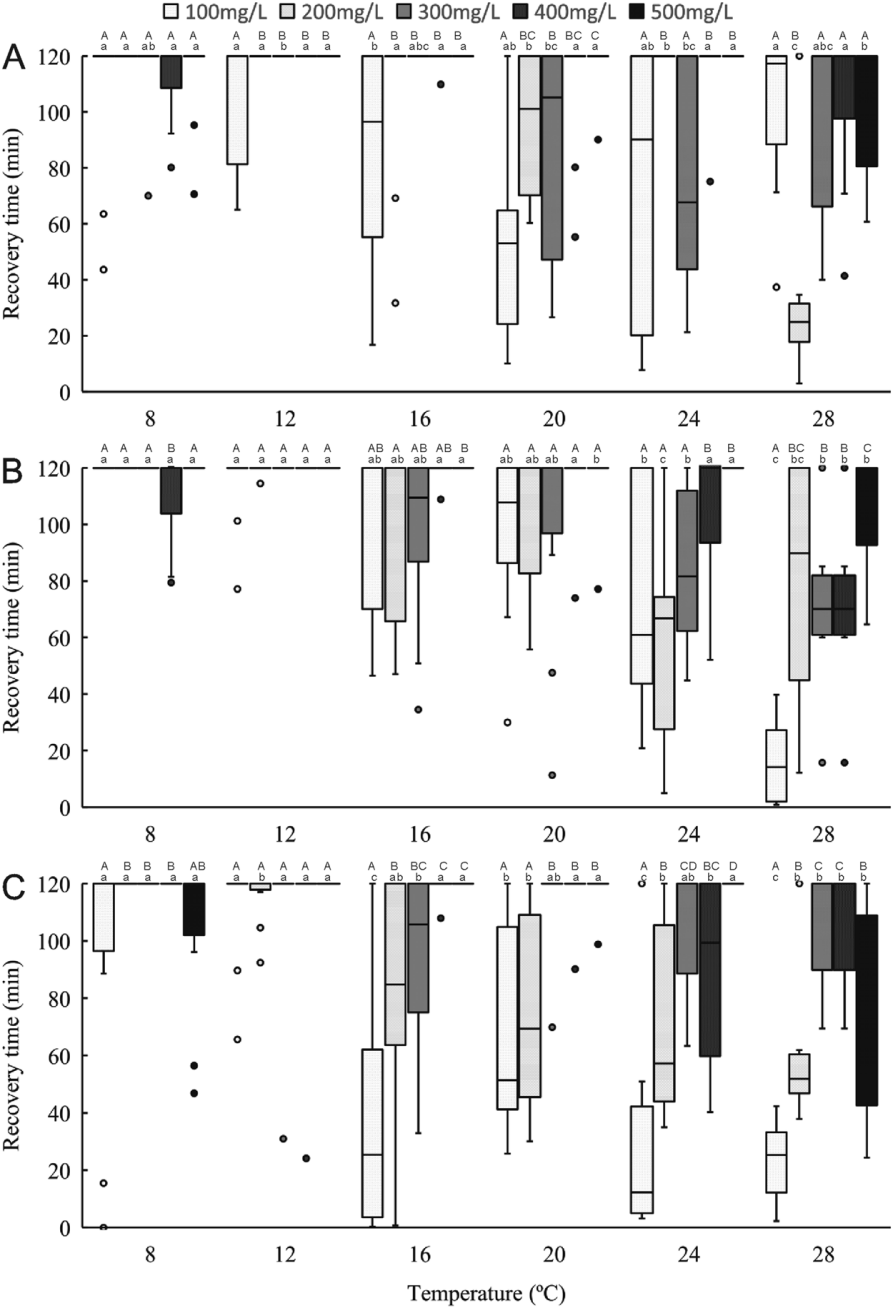
The time required for anaesthetic recovery stage 2 in different eugenol concentritiaons and tempertures through small (**A**), normal (**B**) and large (**C**) shrimps One-way ANOVA showed significant (*P* < 0.05) temperature and dose effects for each size of *Palaemonetes sinensis*. Lowercase letters indicate significant differences within a temperature. Uppercase letters indicate significant differences within a dose.

Figures 3 and 4 show the time to recovery stages 1 and 2 for all size classes and concentrations. Water temperature, eugenol concentration, size class, and their interactions significantly affected recovery time (*P* < 0.05) (Table S1). Similar to induction time, recovery time was less variable at 500 μL/L eugenol than at other concentrations, across all size classes and temperatures. For recovery stage 1, the recovery time of large shrimps increased linearly as concentration and temperature increased. At temperatures of 8 °C and 12 °C, recovery time to stage 2 required more than 2 h for all sizes of shrimp at all concentrations.

### Weight and survival

Percentage weight changes of shrimps after induction in response to different temperatures and eugenol concentrations are shown in Table 1. The mean weight of shrimps was higher after induction at 12 °C, 16 °C, and 20 °C and 100–300 μL/L eugenol. At temperatures of 24 °C and 28 °C, all size classes of shrimp showed a significant decrease in weight after induction, especially at 400 and 500 μL/L eugenol.

**Table 1:** Mean wet weight changes (%) for three sizes of *Palaemonetes sinensis* (Sollaud, 1911) at six temperatures and five doses of eugenol after anaesthesia.

Temperature (°C)	Wet weight change (%)														
	Small					Medium					Large				
	100 μl/L	200 μl/L	300 μl/L	400 μl/L	500 μl/L	100 μl/L	200 μl/L	300 μl/L	400 μl/L	500 μl/L	100 μl/L	200 μl/L	300 μl/L	400 μl/L	500 μl/L
8	−1.50	−5.19	−3.39	−0.71	3.79	2.44	−8.51	1.33	2.70	−5.66	2.27	0.26	−5.18	−2.96	1.90
12	−0.85	5.50	−6.35	7.30	5.88	13.17	2.67	−4.35	8.25	−1.68	−2.65	−4.02	1.84	−8.88	−4.12
16	3.28	5.69	1.67	−6.61	−0.78	3.39	−11.59	0.49	2.72	4.11	−0.45	−3.79	1.84	0.77	−6.56
20	−5.17	9.26	0.89	7.14	−2.68	1.12	0.60	0.00	−2.35	5.19	4.84	2.87	−4.24	−4.80	2.12
24	−11.67	−16.00	−17.19	−2.61	−1.89	−3.77	−14.55	−5.66	−2.63	−2.35	−7.99	−5.50	13.57	−6.87	−2.13
28	−1.51	−13.85	−11.22	−11.86	−2.88	−4.62	−9.96	−0.97	−9.96	−3.81	−0.23	−7.87	−7.48	−7.85	−9.56

Five-day survival data of shrimps after anaesthesia are summarized in Table 2. At 100 and 200 μL/L eugenol concentrations, the survival rates of medium and large shrimps were above 80% over 5 days at all temperatures tested. However, the survival rates of smaller shrimps were below 60% at temperatures above 20 °C over 5 days of recovery. At 300 μL/L eugenol concentration, the survival rates of shrimps of all sizes at 16 °C and 20 °C were maintained above 70% over 5 days. At eugenol concentrations over 400 μL/L, the highest survival rates were achieved at 20 °C, 20 °C, and 24 °C for small, medium, and large shrimps, respectively.

**Table 2:** Survival rates (%) of 10 individuals in three size of *Palaemonetes sinensis* for the six temperatures at five dose of eugenol in 120 hours.

Dose (μl/L)	Temperature (°C)	Small					Medium					Large				
		24 h	48 h	72 h	96 h	120 h	24 h	48 h	72 h	96 h	120 h	24 h	48 h	72 h	96 h	120 h
100	8	100.00%	100.00%	100.00%	100.00%	100.00%	100.00%	100.00%	100.00%	100.00%	100.00%	100.00%	100.00%	100.00%	100.00%	100.00%
	12	90.00%	90.00%	90.00%	90.00%	90.00%	100.00%	100.00%	90.00%	80.00%	70.00%	100.00%	100.00%	100.00%	100.00%	100.00%
	16	90.00%	90.00%	90.00%	90.00%	90.00%	100.00%	100.00%	90.00%	80.00%	80.00%	90.00%	90.00%	90.00%	90.00%	90.00%
	20	80.00%	80.00%	80.00%	80.00%	80.00%	100.00%	100.00%	100.00%	100.00%	100.00%	100.00%	100.00%	100.00%	100.00%	100.00%
	24	60.00%	60.00%	60.00%	60.00%	30.00%	100.00%	90.00%	90.00%	80.00%	70.00%	90.00%	90.00%	90.00%	90.00%	80.00%
	28	50.00%	50.00%	50.00%	50.00%	50.00%	100.00%	100.00%	100.00%	100.00%	90.00%	100.00%	90.00%	90.00%	90.00%	90.00%
200	8	100.00%	100.00%	100.00%	100.00%	100.00%	100.00%	100.00%	100.00%	100.00%	100.00%	100.00%	100.00%	100.00%	100.00%	100.00%
	12	90.00%	90.00%	90.00%	80.00%	80.00%	100.00%	100.00%	100.00%	90.00%	80.00%	90.00%	90.00%	90.00%	90.00%	90.00%
	16	80.00%	80.00%	70.00%	70.00%	70.00%	100.00%	90.00%	90.00%	90.00%	80.00%	100.00%	100.00%	100.00%	100.00%	100.00%
	20	60.00%	60.00%	60.00%	60.00%	50.00%	90.00%	80.00%	80.00%	80.00%	80.00%	100.00%	100.00%	100.00%	100.00%	100.00%
	24	60.00%	30.00%	30.00%	30.00%	30.00%	90.00%	90.00%	90.00%	90.00%	90.00%	90.00%	90.00%	90.00%	90.00%	90.00%
	28	30.00%	30.00%	30.00%	30.00%	30.00%	80.00%	80.00%	80.00%	80.00%	80.00%	100.00%	100.00%	100.00%	100.00%	90.00%
300	8	80.00%	70.00%	70.00%	60.00%	60.00%	70.00%	70.00%	70.00%	70.00%	70.00%	60.00%	60.00%	60.00%	60.00%	60.00%
	12	50.00%	50.00%	50.00%	50.00%	50.00%	50.00%	50.00%	50.00%	50.00%	50.00%	80.00%	70.00%	70.00%	70.00%	70.00%
	16	70.00%	70.00%	70.00%	70.00%	70.00%	100.00%	100.00%	100.00%	90.00%	90.00%	100.00%	100.00%	100.00%	90.00%	90.00%
	20	80.00%	70.00%	70.00%	70.00%	70.00%	70.00%	70.00%	70.00%	70.00%	70.00%	100.00%	100.00%	100.00%	100.00%	100.00%
	24	70.00%	70.00%	50.00%	50.00%	50.00%	90.00%	80.00%	80.00%	80.00%	80.00%	60.00%	60.00%	60.00%	60.00%	60.00%
	28	40.00%	30.00%	30.00%	20.00%	20.00%	90.00%	90.00%	90.00%	80.00%	80.00%	90.00%	90.00%	70.00%	70.00%	70.00%
400	8	100.00%	100.00%	100.00%	100.00%	100.00%	90.00%	90.00%	80.00%	80.00%	80.00%	60.00%	60.00%	60.00%	60.00%	60.00%
	12	40.00%	40.00%	40.00%	40.00%	40.00%	60.00%	60.00%	60.00%	60.00%	60.00%	60.00%	60.00%	60.00%	60.00%	60.00%
	16	30.00%	30.00%	30.00%	30.00%	30.00%	10.00%	10.00%	10.00%	10.00%	10.00%	70.00%	70.00%	70.00%	70.00%	70.00%
	20	80.00%	80.00%	80.00%	80.00%	70.00%	80.00%	80.00%	80.00%	80.00%	80.00%	70.00%	70.00%	60.00%	60.00%	60.00%
	24	30.00%	30.00%	20.00%	20.00%	20.00%	70.00%	70.00%	70.00%	70.00%	70.00%	80.00%	80.00%	70.00%	70.00%	70.00%
	28	30.00%	30.00%	30.00%	30.00%	30.00%	90.00%	80.00%	80.00%	80.00%	80.00%	60.00%	40.00%	40.00%	40.00%	40.00%
500	8	50.00%	50.00%	50.00%	50.00%	50.00%	80.00%	80.00%	80.00%	80.00%	80.00%	60.00%	60.00%	50.00%	50.00%	50.00%
	12	40.00%	30.00%	30.00%	30.00%	30.00%	50.00%	40.00%	40.00%	40.00%	40.00%	20.00%	20.00%	20.00%	20.00%	20.00%
	16	20.00%	20.00%	20.00%	20.00%	20.00%	40.00%	30.00%	30.00%	30.00%	30.00%	40.00%	30.00%	30.00%	30.00%	30.00%
	20	70.00%	70.00%	70.00%	70.00%	70.00%	50.00%	50.00%	50.00%	50.00%	50.00%	30.00%	30.00%	30.00%	30.00%	30.00%
	24	30.00%	30.00%	30.00%	30.00%	30.00%	50.00%	50.00%	50.00%	50.00%	50.00%	10.00%	10.00%	10.00%	10.00%	10.00%
	28	60.00%	40.00%	30.00%	30.00%	30.00%	80.00%	80.00%	30.00%	30.00%	30.00%	70.00%	70.00%	30.00%	30.00%	30.00%

## Discussion

Anaesthetics such as MS-222 and Aqui-S^M^ are approved for food-safe use in aquaculture animals in most countries, including the USA, Australia, New Zealand, and countries of the European Union. However, it seems that considerably higher concentrations are required to anaesthetise crustaceans than those required for fish^18,19^. Carbon dioxide and cooling are effective for most crustaceans, but such approaches are expensive and inconvenient. To date, there are no recommended anaesthetics for crustaceans owing to the lack of such studies. Essential oils such as eugenol and menthol are being used as cheap and safe anaesthetics in many crustaceans in recent years. Eugenol is easily obtained and is generally considered safe by most national authorities. It has also proved effective in many crustaceans, including *Homarus americanus*^12^, *Fenneropenaeus indicus*^20^, *Macrobrachium rosenbergii*^1^, and *Nephrops norvegicus*^21^. In the present study, eugenol was found to be effective for anaesthetising *P. sinensis*. Moreover, the price of eugenol is about 40 US dollars per kilogram, which is cheaper than most commercial aquatic anaesthetics, such as MS-222, benzocaine, quinaldine, 2-phenoxyethanol, metomidate and Aqui-S^TM^ (data from Liyang Aquatic Technology Co., Ltd, Guangzhou, China).

Various factors such as body size^22,23^, water temperature^24^, and anaesthetic dose^25^ have a significant effect on anaesthesia in fishes. However, the relationships among these factors and anaesthesia in crustaceans have not been investigated before. Our results indicate that induction and recovery times for *P. sinensis* are related to its size, in that both small and medium-sized shrimps can be induced to anaesthesia stages 1 and 2 in 60 min at any temperature and or dosage, while larger shrimps require a significantly higher induction time (*P* < 0.05), especially at lower temperatures and doses. Similar findings have been reported for the angelfish *Pterophyllum scalare*^22^ and the iridescent shark *Pangasius hypophthalmus*^26^. This might be because the gill surface of crustaceans is correlated with body size^27^, leading to higher rates of anaesthetic uptake through the gills in smaller shrimps compared to larger ones. Furthermore, small shrimps suffered higher mortality than medium and large shrimps, and mortality also increased with dose and temperature, perhaps owing to a greater surface area to volume ratio, leading to faster anaesthetic absorption.

Five temperatures from 8 °C to 28 °C were investigated in this study. The induction and recovery times for anaesthesia were greater at low temperatures than at high temperatures, comparable to reports from fish and molluscs^23,28,29^. Above 20 °C, the time to induction stage 1 for all groups was less than 40 min, but caused higher mortality, especially at concentrations above 300 μL/L eugenol. However, shrimps at low temperature (8 °C) took a long time to reach anaesthesia stage 2 under 100 μL/L eugenol, and the interaction of temperature and size had significant effects on both induction and recovery times (*P* < 0.05). It is widely hypothesized that the uptake of an anaesthetic across the respiratory and circulatory system might be fast enough that the interaction effects of temperature and size are minimized^3,30^. However, here, the anaesthetic efficiency was not significantly affected by the interaction of temperature and dose at induction stage 1 (*P* > 0.05).

Although the efficacy of eugenol as an anaesthetic has been studied in some crustacean species, its safe and effective concentrations have not been clearly defined. In the present study, concentrations ranging from 100 to 500 μL/L were all found to be effective in immobilizing shrimps of different size classes and temperature conditions. Although induction times for *P. sinensis* were significantly shorter at higher concentrations (*P* < 0.05), the safe dose of eugenol was found to be 200 μL/L, which yielded adequate anaesthesia and ensured optimised survival over a 5-day recovery period. This indicates that this dose provides effective results with an appropriate margin of safety.

In conclusion, we found that eugenol is an effective anaesthetic for potential use in the Chinese grass shrimp *P. sinensis*. This will be useful in commercial applications such as handling, netting, and long-distance live transport, which are difficult and expensive for many crustacean species^19,20^. The results of this experiment can be used to determine the effectiveness of different concentrations for different size classes during the capture and transport process from wild environments. Especially for *P. sinensis*, a typical r-selected specie that can be netted and transported all the year round. Higher temperatures produced a rapid uniform response in all size classes but were associated with high morality at eugenol concentrations over 400 μL/L; therefore, high-dose eugenol is not recommended for use in summer. In winter, when water temperature is below 16 °C, eugenol at concentrations of 100–300 μL/L can be used effectively and safely for all size shrimps anaesthetised for holding or transport. However, the effects of certain environmental factors such as salinity, pH, and oxygen levels, as well as biological factors such as body condition, developmental stage, and lipid content, were not tested in this study. Further research is recommended to focus on these, as well as on the long-term health effects of anaesthesia, such as stress responses, immune responses, pharmacokinetics, and metabolism.

## Materials and Methods

### Shrimps

Approximately 15,000 individuals (11 kg) of *P. sinensis* were used in this study. The shrimps were collected from a rice field in Panjin City, Liaoning Province, China, in October 2016, and then transported to the aquaculture laboratory in Shenyang Agricultural University. They were distributed equally into 30 tanks (300 L capacity) with a circular flow system at 18 °C ± 0.5 °C and a 12:12 h light:dark cycle, and held for 30 days prior to experimentation. Every 10 tanks were connected to a biological filter tank, temperature-controlling device, and UV light device. Aeration was provided by a Roots-type blower and air stones. Freshwater oligochaetes were offered as food at a rate of 2% of body weight every 24 h.

After the 30-day period elapsed, every 5 tanks of the 30 tanks were acclimated and maintained for 2 weeks at 8 °C, 12 °C, 16 °C, 20 °C, 24 °C, and 28 °C, with 1 °C rise per day until the target temperature was reached. After acclimatisation, all shrimps were starved for 24 h prior to anaesthetic experimentation. Shrimps in poor physical condition were excluded from further experimental procedures.

### Anaesthetic preparation and experimental design

Eugenol (500 mL, 99% purity) was purchased from Jiangxi Xuesong Natural Medicinal Oil Co., Ltd. (Jian City, Jiangxi Province, China) and dissolved in 95% ethanol at a 1:10 v:v ratio. Five different concentrations of eugenol (100, 200, 300, 400, and 500 μL/L) were formulated according to preliminary studies on *Fenneropenaeus indicus*^13^ and *Litopenaeus vannamei*^31^.

Shrimps were parsed into size groups according to body length as follows: small (1.5–2.5 cm), medium (2.5–3.5 cm), and large (3.5–4.5 cm). Ten shrimps of each group were held in five separate plastic tanks (5 L capacity) containing different concentrations of eugenol (100, 200, 300, 400, and 500 μL/L). Water used in the experiment was supplied from the acclimatisation tanks, and temperature was maintained by using six constant-temperature incubators (GXZ-500; Ningbojiangnan Co. Ltd., Ningbo City, Zhejiang Province, China). The four stages of induction and recovery from anaesthesia under identical experimental conditions are described in Table 3^17,18^. The time required to reach each stage was recorded by one observer per stage. After shrimps showed a complete loss of equilibrium and non-reactivity to stimuli (induction stage 2), they were removed from the anaesthetic tank to a recovery tank (20 × 40 × 30 cm) using a hand net. The recovery tanks were filled with 10 L tap water at 20 °C ± 1 °C. Shrimps were considered eugenol free when they reached recovery stage 2. The survival rate for each recovery tank was measured every 24 h over the subsequent 5 days. Change in wet weight was calculated based on the body weight of each individual measured before and after induction. These experiments were repeated at 8 °C, 12 °C, 16 °C, 20 °C, 24 °C, and 28 °C. Each shrimp was used only once, and for a maximum time of 120 min.

**Table 3:** Stages of anaesthesia and recovery in shrimps.

Stage	Description	Characteristic behaviour
*Induction*		
1	Partial loss of equilibrium	Reaction only to strong tactile and vibration stimuli
2	Complete loss of equilibrium	Percular movement ceases, not reactive to stimuli
*Recovery*		
1	Partial regained control of equilibrium	Start of erratic swimming without reestablishment of equilibrium
2	Complete regained control of equilibrium	Attained an upright position on the bottoms of the aquaria

### Statistical analysis

Box plots were produced based on induction and recovery times for each size class of shrimp at each temperature and concentration, using Microsoft Excel. One-way and three-way ANOVAs were used to detect differences in induction and recovery times among size class, temperature, and concentration, and significant differences were determined by Duncan’s post-hoc test. The significance level for all analyses was set at P < 0.05. Statistical analyses were carried out using SPSS 17.0 software.

### Ethics statement

Our study did not involve endangered or protected species. In China, catching wild grass shrimp from rice fields does not require specific permits. Animal welfare and the relevant experiment were carried out in compliance with the guide for the care and use of laboratory animals. The experimental protocol was approved by the Animal Ethics Committee of Shenyang Agriculture University.

## Electronic supplementary material

Supplementary Material
