# Supplementary material for: Anaesthetic Effects of Eugenol on Grass Shrimp (Palaemonetes sinensis) of Different Sizes at Different Concentrations and Temperatures
Source: Sci Rep. 2018 Jul 20;8:11007. doi: 10.1038/s41598-018-28975-w (PMC6054647; doi:10.1038/s41598-018-28975-w)
Supplement: Supplementary file 1 — Supplementary Material [file 41598_2018_28975_MOESM1_ESM.doc]

**1.Title of manuscript:**

Anaesthetic Effects of Eugenol on Grass Shrimp (*Palaemonetes sinensis*) of Different Sizes at Different Concentrations and Temperatures

**2. Author list:**

| Author | Salutation | Given name | Family name | Email |
| --- | --- | --- | --- | --- |
| First author | Dr. | Yingdong | Li | liyingdong@syau.edu.cn |
| Second author | Mr. | Zhibin | Han |  |
| Third author | Ms. | Qiuxin | She |  |
| Fourth author | Ms. | Na | Sun |  |
| Fifth author | Mr. | Xu | Liu |  |
| Sixth author/Corresponding author | Prof. | Xiaodong | Li | lxd001@ceraap.com |

Table S1 Three-way ANOVA results for the effect of temperature, size, dose and their interaction on anaesthetic efficacy in *Palaemonetes sinensis*

| *Stage* | *Univariate Model* | *Factors&Covariates* | d.f.s | ANOVA SS | Mean square | *P*-value |
| --- | --- | --- | --- | --- | --- | --- |
| Anaesthesia stage 1 | Main effects | Dose | 5 | 776.5 | 10.4 | <0.0001 |
|  |  | Temperature | 4 | 4383.9 | 58.5 | <0.0001 |
|  |  | Size | 2 | 1438.1 | 19.2 | <0.0001 |
|  | Interaction | Temperature*Dose | 20 | 80.5 | 1.2 | 0.258 |
|  |  | Dose*Size | 10 | 330.0 | 4.9 | <0.0001 |
|  |  | Temperature*Size | 8 | 497.9 | 7.3 | <0.0001 |
|  |  | Temperature*Size*Dose | 89 | 443.6 | 7.0 | <0.0001 |
| Anaesthesia stage 2 | Main factor | Dose | 5 | 3463.7 | 22.1 | <0.0001 |
|  |  | Temperature | 4 | 21071.1 | 134.3 | <0.0001 |
|  |  | Size | 2 | 10451.8 | 66.6 | <0.0001 |
|  | Interaction | Temperature*Dose | 20 | 808.1 | 7.0 | <0.0001 |
|  |  | Dose*Size | 10 | 529.3 | 4.6 | <0.0001 |
|  |  | Temperature*Size | 8 | 2421.2 | 20.9 | <0.0001 |
|  |  | Temperature*Size*Dose | 89 | 2023.1 | 20.0 | <0.0001 |
| Recovery stage 1 | Main factor | Dose | 5 | 37784.1 | 22.2 | <0.0001 |
|  |  | Temperature | 4 | 81054.9 | 47.7 | <0.0001 |
|  |  | Size | 2 | 35231.9 | 20.7 | <0.0001 |
|  | Interaction | Temperature*Dose | 20 | 6622.2 | 4.3 | <0.0001 |
|  |  | Dose*Size | 10 | 2456.6 | 1.6 | 0.107 |
|  |  | Temperature*Size | 8 | 4073.0 | 2.6 | 0.008 |
|  |  | Temperature*Size*Dose | 89 | 10202.6 | 7.0 | <0.0001 |
| Recovery stage 2 | Main factor | Dose | 5 | 34021.4 | 42.2 | <0.0001 |
|  |  | Temperature | 4 | 36566.4 | 45.3 | <0.0001 |
|  |  | Size | 2 | 5400.8 | 6.7 | 0.001 |
|  | Interaction | Temperature*Dose | 20 | 3274.4 | 4.6 | <0.0001 |
|  |  | Dose*Size | 10 | 2222.6 | 3.1 | 0.001 |
|  |  | Temperature*Size | 8 | 3385.8 | 4.8 | <0.0001 |
|  |  | Temperature*Size*Dose | 89 | 6238.1 | 10.3 | <0.0001 |
